# Individual and organizational predictors of allied healthcare providers’ job satisfaction in residential long-term care

**DOI:** 10.1186/s12913-018-3307-3

**Published:** 2018-06-25

**Authors:** Laura D. Aloisio, Wendy A. Gifford, Katherine S. McGilton, Michelle Lalonde, Carole A. Estabrooks, Janet E. Squires

**Affiliations:** 1 School of Nursing, University of Ottawa, Roger Guindon Hall Room 3051, 451 Smyth Road, Ottawa, ON K1H 8M5 Canada; 2 Toronto Rehabilitation Institute – University Health Network, Lawrence S Bloomberg - Faculty of Nursing, University of Toronto, 133 Dunn Ave, Toronto, ON M6K 2R7 Canada; 3 School of Nursing, University of Ottawa, Roger Guindon Hall Room 3249B, 451 Smyth Road, Ottawa, ON K1H 8M5 Canada; 4 Faculty of Nursing, Edmonton Clinic Health Academy, University of Alberta, 11405-87 Avenue, Edmonton, AB T6G 1C9 Canada; 5 Ottawa Hospital Research Institute, General Campus, 501 Smyth Rd, Box 711, Ottawa, ON K1H 8L6 Canada

**Keywords:** Job satisfaction, Allied health professionals, Long-term care, Individual-level factors, Context-level factors, Work environment

## Abstract

**Background:**

Job satisfaction is a predictor of intention to stay and turnover among allied healthcare providers. However, there is limited research examining job satisfaction among allied health professionals, specifically in residential long-term care (LTC) settings. The purpose of this study was to identify factors (demographic, individual, and organizational) that predict job satisfaction among allied healthcare providers in residential LTC.

**Methods:**

We conducted a secondary analysis of data from Phase 2 of the Translating Research in Elder Care program. A total of 334 allied healthcare providers from 77 residential LTC in three Western Canadian provinces were included in the analysis. Generalized estimating equation modeling was used to assess demographics, individual, and organizational context predictors of allied healthcare providers’ job satisfaction. We measured job satisfaction using the Michigan Organizational Assessment Questionnaire Job Satisfaction Subscale.

**Results:**

Both individual and organizational context variables predicted job satisfaction among allied healthcare providers employed in LTC. Demographic variables did not predict job satisfaction. At the individual level, burnout (cynicism) (β = −.113, *p* = .001) and the competence subscale of psychological empowerment (β = −.224, *p* = < .001), were predictive of lower job satisfaction levels while higher scores on the meaning (β = .232, *p* = .001), self-determination (β = .128, *p* = .005), and impact (β = .10, *p* = .014) subscales of psychological empowerment predicted higher job satisfaction. Organizational context variables that predicted job satisfaction included: social capital (β = .158, *p* = .012), organizational slack-time (β = .096, *p* = .029), and adequate orientation (β = .088, *p* = .005).

**Conclusions:**

This study suggests that individual allied healthcare provider and organizational context features are both predictive of allied healthcare provider job satisfaction in residential LTC settings. Unlike demographics and structural characteristics of LTC facilities, all variables identified as important to allied healthcare providers’ job satisfaction in this study are potentially modifiable, and therefore amenable to intervention.

**Electronic supplementary material:**

The online version of this article (10.1186/s12913-018-3307-3) contains supplementary material, which is available to authorized users.

## Background

In 2014, approximately 2.6% of seniors 65 years and over and 9.5% of seniors 85 years and over resided in long-term care (LTC) facilities in the United States [1]. In Canada, in 2011, approximately 224,280 seniors reside in LTC facilities [2]. Within LTC facilities, allied healthcare providers (e.g., social workers, physiotherapists and aides, psychologists, dietitians, recreation therapists and aides) work together with nursing staff (e.g., registered nurses, licensed practical nurses, healthcare aides) to meet residents’ needs and ensure a high quality of care. Job satisfaction among LTC care providers however is suboptimal [3] and has been shown to adversely affect the quality of care provided to residents [4, 5] by increasing turnover [6–12], reducing employees’ well-being [8], increasing risk of medical errors [8], and increasing risk of adverse health events [13–15].

The factors affecting job satisfaction, specifically among allied healthcare providers employed in LTC facilities, are poorly understood. Previous studies have found limited empirical support for a relationship between demographic factors and job satisfaction [16–19]. Among individual-level factors, psychological empowerment [16–18] and physical and mental health [18] are shown to be related to allied healthcare provider job satisfaction in LTC. There is also some support for organizational context variables, namely: leadership and supervisory support (the actions of leaders in an organization to influence change and practice [16, 18]), culture (the way things are done in organizations and work units [16, 18]), and social capital (the stock of active connections among people [16]). Overall, there is a paucity of research on allied healthcare providers’ job satisfaction in LTC, particularly in Canadian LTC settings. Given allied healthcare providers’ role in providing high quality resident care in LTC, it is important that we know the factors, individual and organizational, that affect their job satisfaction. Therefore, the purpose of this study was to identify factors that predict job satisfaction among allied healthcare providers in residential LTC.

### Theoretical framework

Kanter’s [20] Theory of Structural Empowerment and Spreitzer’s [21–23] Theory of Psychological Empowerment were used to guide our identification and understanding of the factors important to allied healthcare provider job satisfaction [24]. Focusing on the role of organizational context, Kanter’s [20] Theory identifies three organizational factors that affect employees’ job satisfaction: 1) opportunity for professional development and advancement; 2) access to resources required for meeting professional and organizational goals; and 3) structure of power, which includes access to information and support, guidance, and feedback from others [20]. Kanter [20] posited that employees who receive adequate support and have access to resources, opportunities for professional development, and higher levels of formal and informal power exhibit higher levels of work satisfaction. Focusing on how employees perceive their work environment, Spreitzer’s [21, 22] Theory of Psychological Empowerment posited that psychological empowerment, which concerns itself with employees’ intrinsic motivations [25], is necessary for job satisfaction. Spreitzer [21, 22] argued that four components of psychological empowerment—competence, meaning, self-determination, and impact—are important to ensure employees experience psychological empowerment, which in turn promotes job satisfaction. Consistent with the most recent findings in the literature, we felt that the combination of both theories offered a better understanding of job satisfaction [26–30].

#### Kanter’s theory of structural empowerment

Within healthcare, Kanter’s theory has historically most often been applied to the study of nurses [26, 31–34]. More recently, studies have begun using Kanter’s theory to examine the effects of structural empowerment on job commitment, job satisfaction, burnout, and turnover intention in allied health professionals [35–40]. For instance, Gilbert, Laschinger, and Leiter [35] used Kanter’s theory to examine how structural empowerment affects healthcare professionals’ use of “organizational citizenship behaviours” [35] in different hospital units. Similarly, in a study including nursing assistants, nurses’ aides, registered nurses, physiotherapists, and occupational therapists employed in nursing homes or home-help services in Sweden, Hagerman et al. [36] used Kanter’s theory to examine the relationship between empowerment, stress, and performance. Kanter’s theory has also been applied to examine structural empowerment on job commitment and turnover intention among pharmacists [37], and physical therapists [38, 40], physiotherapists, and occupational therapists [40]. Miller, Goddard, and Laschinger [38] found evidence in support of Kanter’s theory as a framework to examine factors important in empowering physical therapists. However, these studies have not directly examined whether structural empowerment enhances job satisfaction among allied health workers.

#### Spreitzer’s theory of psychological empowerment

In the Theory of Psychological Empowerment, Spreitzer [21, 22] was concerned with how employees interpret their work environment psychologically. Psychological empowerment refers to a state experienced by employees that is required for employers’ interventions to succeed [21, 22]. There are four components of psychological empowerment: 1) competence (employee’s confidence in their ability to do their job); 2) meaning (compatibility between an employee’s value and beliefs and the requirements of the job); 3) self-determination (feelings of control over their place of employment); and 4) impact (a sense that one’s contributions are important and meaningful to the organization) [21, 22]. According to Spreitzer, high levels of psychological empowerment promote job satisfaction.

Each of the four dimensions of Spreitzer’s theoretical framework have been studied and found to be associated with job satisfaction; however, as with Kanter’s theory, most of the research on this area has examined the applicability of Spreitzer’s theory in a nursing context [31–33, 41, 42]. In nursing studies, organizational empowerment predicted job satisfaction among nurse educators [33], hospital nursing staff [42], and staff nurses in critical care, med-surgical, and maternal-child units [41]. Among nurse educators, Sarmiento et al. [33] found that opportunity for advancement, access to information, access to resources, support, and formal and informal power were significantly related to overall job satisfaction.

Spreitzer’s theory has also been applied to health care contexts more broadly. For instance, a study by Wagner et al. [39] of over 5000 health organization employees found that psychological empowerment predicts job satisfaction (and turnover intention). Wagner et al. [39] concluded that their findings provide strong support for the role of psychological empowerment as a mediator between organizational context factors and job satisfaction.

#### Relationship between psychological & structural empowerment

More recently, researchers have begun integrating theories of structural and psychological empowerment to gain a more comprehensive understanding of the factors affecting job satisfaction [43]. Researchers have found that both structural and psychological empowerment correlate with job satisfaction in nurses [29, 30] but that the combination of both theories more strongly predicts job satisfaction than when used alone [27]. In nurses, psychological empowerment has also been shown to mediate the relationship between structural empowerment [26] and job satisfaction, as well as workplace environment and job satisfaction [28]. These studies provide support for the notion that organizational empowerment affects employees’ job satisfaction by promoting psychological empowerment [26–30]. Though in nurses, this conclusion was reached in a recent review by Cicolini, Comparcini, and Simonetti [44], who found that psychological empowerment mediated the relationship between structural empowerment and job satisfaction [44]. More recently, studies have begun examining the relationship between these theories and job satisfaction among allied health workers. For example, Wagner et al. [40] investigated the role of structural empowerment and psychological empowerment on job satisfaction among physical therapy and occupational therapy practitioners’ perceptions of resonant leadership [40]. Taken together, these findings provide support for the role of structural and psychological empowerment in employees’ job satisfaction.

## Methods

### Design

An analysis was performed on data collected from Phase 2 of the Translating Research in Elder Care (TREC) program. TREC is a multi-level, descriptive, longitudinal research program whose aim is to develop a comprehensive understanding of the effect of organizational context on research utilization, and the impact of research utilization on care providers’ use of best practices, quality of work life, and staff and resident health outcomes in residential LTC facilities [45]. The eventual goal is to utilize this knowledge to improve quality of care, resident health outcomes, and work life among LTC management and staff.

We sought to utilize TREC data because its breadth and depth made it possible for us to perform a secondary analysis to examine factors, including psychological empowerment, affecting job satisfaction among allied health staff. Specifically, the rationale for utilizing TREC data was threefold: (1) the same surveys were administered to all allied health staff within residential LTC facilities; (2) the TREC survey was designed to assess quality of work-life outcomes, including job satisfaction; and (3) factors from demographic, individual-level, and context-level that have been shown to be related to and/or are theoretically important to job satisfaction were captured. Additionally, TREC data was successfully used to determine several individual and organizational factors contributing to job satisfaction among healthcare aides [46]. Using the TREC data provided us with a unique opportunity to compare and contrast factors that are empirically or theoretically related to job satisfaction in allied health staff in LTC settings.

### Sampling

TREC maintains a longitudinal database with waves of primary data collection every 2–3 years coupled with regular acquisition of routinely collected resident data. Presently, the TREC database contains information collected from 77 LTC facilities in British Columbia, Alberta, and Manitoba. Facilities were selected using stratified random sampling by health region within the three provinces, owner-operator model (public not-for-profit; voluntary not-for-profit; private for-profit), and size (small: ≤79 beds, medium: 80–120 beds, large: > 120 beds) [45].

### Data sources and collection

In each province, a local team led by a site investigator was responsible for recruitment and data collection. Although data was collected from healthcare aides (unregulated workers), regulated nurses (RNs and LPNs), allied health staff (including social workers, dieticians, pharmacists, rehabilitation therapists, recreation therapists), physicians, practice specialists, and care managers, only data collected from allied health staff was analyzed in the present study. The allied health provider data used in this analysis was collected using web-based surveys between September 2014 and May 2015. Participants were recruited using a volunteer, census-like sampling technique. Diverse communication strategies were used to inform participants about the study. All allied healthcare providers in the 77 TREC LTC facilities who identified themselves under the broad category of “allied staff” were handed a package that included an access code to complete the TREC allied healthcare provider survey online. Staff was not asked which subcategory of allied staff they fell under until they were asked to identify their role in the survey. Eligibility criteria included working at the facility in their current role for at least 3 months and working a minimum of six shifts per month. A total of 721 packages were handed out, and 334 surveys were successfully completed by eligible participants (overall response rate: 46.3%). Reason for non-participation was not provided.

### Measures

The TREC allied healthcare provider survey measured: demographics (e.g. gender), individual factors (including health outcomes, e.g. physical and mental health status, and work life outcomes, e.g. empowerment), and organizational context variables (e.g. leadership). The TREC facility survey included three variables: owner model, size, and province. All survey variables used in the analysis presented in this paper along with their definitions, measurement, and reliability are listed in Table 1.

**Table 1:** Descriptions of variables in TREC 2.0 relevant to present study

Category	Variable	Definition	Measurement	Cronbach’s Alpha
Demographic Characteristics	Gender	An individual’s gender	Asked for their gender: male or female	N/A
	Age group	An individual’s age group	Asked to indicate age group according to a category (< 20 years, 20–29, 30–39, etc.)	N/A
	Highest Education	Level of education obtained	Asked to answer yes or no if completed diploma/certificate, bachelor’s, or graduate degree	N/A
	Current Enrolment Status	Current enrolment in an educational program	Asked to answer: yes or no	N/A
	Years Worked in Current Role	Total years in current role	Asked for number of years and months worked in current role	N/A
	Years Worked in Facility	Total years worked on unit	Asked for number of years and months worked on unit	N/A
	Hours Worked	Average hours worked per week during a two-week period	Asked for number of hours in last typical two-week period	N/A
	Year of Licensure	Year the individual became licensed	Asked to indicate year of licensure	N/A
	Primary Role	An individual’s primary role	Asked to select their primary role: rehabilitation therapist, clinical pharmacist, recreational therapist, social worker, dietician, rehabilitation therapist assistant/attendant/aid, recreational therapist assistant/attendant/aide, other	N/A
Individual-Level Factors	Burnout-Emotional Exhaustion	A debilitating psychological condition brought about by unrelieved work stress	Maslach Burnout Inventory General Survey (short form) [48]. 3 items per subscale scored on a 7-point Likert frequency scale (never to daily). An overall score for each subscale is derived by taking the mean of 3 items	.806
	Burnout-Cynicism			.655
	Burnout-Efficacy			.674
	Job satisfaction*	An individual’s perception of whether they are “satisfied” with their job	Michigan Organizational Assessment Questionnaire, Job Satisfaction Subscale [47]. 3 items scored on a 5-point Likert agreement scale ranging from strongly disagree (1) to strongly agree (5). An overall score is derived by taking the mean of 3 items	.821
	Work Engagement-Vigor	Work engagement refers to positive, fulfilling work-related state of mind, based on three subscales: vigor, dedication, and absorption	Utrecht Work Engagement Scale 9 [49]. 3 items per subscale scored on a 7-point frequency rating scale ranging from 0 (never) to 6 (daily). An overall score for each subscale is derived by taking the mean of 3 items	.814
	Work Engagement-Dedication			.881
	Work Engagement-Absorption			.751
	Empowerment-Competence	Empowerment is defined as a motivational construct manifested in four cognitions: meaning, competence, self-determination, and impact, which reflects an active as opposed to passive attitude toward work	Spreitzer’s Psychological Empowerment scale [23]. 3 items per subscale scored on a 5-point Likert agreement scale, ranging from strongly disagree (1) to strongly agree (5). An overall score for each subscale is derived by taking the mean of 3 items	.831
	Empowerment-Meaning			.881
	Empowerment-Self-Determination			.931
	Empowerment-Impact			.751
	Problem Solving	The cognitive, affective and behavioural processes and to the particular set of skills people employ in order to find solutions for the challenges of everyday life	Heppner’s Problem Solving Inventory [50]. 10 items scored on a 5-point Likert agreement scale (strongly disagree to strongly agree). An overall score is derived by taking the mean of the 10 items	.543
	Physical Health Status	An individual’s perception of their health status over past 4 weeks	SF-8™ health survey [51]. 8 items scored on 5- or 6-point scales depending on the item. Scoring is done using a proprietary algorithm obtained when permission to use the scale is granted to produce a summary mental and physical health score (0–100%)	.855
	Mental Health Status			.839
Context-Level Factors	ACT: Leadership	Actions of leaders in an organization to influence change and practice; reflect emotionally intelligent leadership	The Alberta Context Tool [52]. 6 items scored on a 5-point Likert agreement scale (strongly disagree to strongly agree). An overall score is derived by taking the mean of the 6 items	.932
	ACT: Culture	The way that “we do things” in our organizations and work units, items reflect a supportive work culture	6 items scored on a 5-point Likert agreement scale (strongly disagree to strongly agree). An overall score is derived by taking the mean of the 6 items	.831
	ACT: Evaluation	The process of using data to assess team performance and to achieve outcomes in organizations or units	6 items scored on a 5-point Likert agreement scale (strongly disagree to strongly agree). An overall score is derived by taking the mean of the 6 items	.919
	ACT: Social Capital	The stock of active connections among people. Connections are of 3 types: bonding, bridging, linking	6 items scored on a 5-point Likert agreement scale (strongly disagree to strongly agree). An overall score is derived by taking the mean of the 6 items	.816
	ACT: Organizational Slack-Staff	The cushion of actual or potential resources which allows an organization (unit) to adapt successfully to internal pressures for adjustments or to external pressures for changes	3 items scored on a 5-point Likert agreement scale (strongly disagree to strongly agree). An overall score is derived by taking the mean of the 3 items	.591
	ACT: Organizational Slack-Time		4 items scored on a 5-point Likert agreement scale (strongly disagree to strongly agree). An overall score is derived by taking the mean of the 4 items	.742
	ACT: Organizational Slack-Space		2 items scored on a 5-point Likert agreement scale (strongly disagree to strongly agree). An overall is derived by taking the mean of the 2 items	.722
	ACT: Formal Interactions	Formal exchanges that occur between individuals working within an organization (unit) through scheduled activities that can promote the transfer of knowledge	4 items scored on a 5-point Likert frequency scale (never to almost always). Recode each of the 4 item scores to “0” (no interaction) and “1” (interaction). An overall score is derived by taking a count of the 4 recoded items	.832
	ACT: Informal Interactions	Informal exchanges that occur between individuals working within an organization (unit) that can promote the transfer of knowledge	9 items scored on a 5-point Likert frequency scale (never to almost always)/ Recode each of the 9 item scores to “0” (no interaction) and “1” (interaction). An overall score is derived by taking a count of the 9 recoded items	.803
	ACT: Structural and Electronic Resources	The structural and electronic elements of an organization (unit) that facilitate the ability to assess and use knowledge	7 items scored on a 5-point Likert frequency scale (never to almost always). Recode each of the 7 item scores to 0 (no resource) or 1 (resource). An overall score is derived by taking a count of the 7 recoded items	.644
	Adequate Orientation	An individual’s perception of whether they have had enough orientation to carry out their job effectively and safely.	A single item scored on a 5-point Likert agreement scale (strongly disagree to strongly agree)	N/A
	Dementia-related Responsive Behaviours	An individual’s experience with violence and difficult behaviours from residents.	Sum of six items: threat of assault, emotional abuse, physical abuse, verbal sexual harassments, sexual assault, and force sexual intercourse. Each item was scored as yes or no based on if the respondent had experienced the behaviour during the last five shifts	N/A
Provincial/Facility Variables	Province	Province in which the residential LTC is located	British Columbia, Alberta, or Manitoba	N/A
	Owner-Operator Model	Ownership and operation model of the facility	Public not-for-profit, private for-profit, or voluntary not-for-profit	N/A
	Facility Size	Total number of beds for residents in the facility	Sum of LTC beds and non-LTC beds (small: ≤79 beds, medium: 80–120 beds, large: > 120 beds)	N/A

Cronbach’s alpha (α): α ≥ 0.9, excellent; 0.9 > α ≥ 0.8, good; 0.8 > α ≥ 0.7, acceptable; 0.7 > α ≥ 0.6, questionable; 0.6 > α ≥ 0.5, poor; 0.5 < α, unacceptable

*Abbreviations*: *ACT* Alberta Context Tool, *LTC* long-term care, *N/A* not applicable as single item

#### Dependent variable

Job satisfaction was defined as a holistic measure of a person’s attitude and feelings toward work. Job satisfaction was measured using the Michigan Organizational Assessment Questionnaire Job Satisfaction Subscale [47], which is a three-item scale, scaled on a five-point Likert scale (1, strongly disagree; 5, strongly agree). An overall score for job satisfaction was produced by taking a mean of the three items (score range 1–5), with higher values representing higher job satisfaction.

#### Independent variables

Independent variables were chosen based on their availability in the TREC allied healthcare provider survey, balanced by the presence of empirical and/or theoretical support in relation to allied healthcare provider job satisfaction. We assessed demographic, individual-level, and organizational context variables.

#### Demographic variables

Demographic variables included: gender, age group, highest education, primary role, current enrolment, hours worked in two weeks, years worked in current role, and years worked in facility. Although previous research studies did not identify demographic factors as predictors of job satisfaction [16–19], we included demographic variables in our bivariate analysis because very few studies have assessed these variables among allied health staff. Thus, we wanted to know if these variables were significant in our population.

#### Individual-level variables

Individual-level variables included: three dimensions of burnout (exhaustion, cynicism, efficacy), measured using the Maslach Burnout Inventory-Short Form [48]; three dimensions of work engagement (vigor, dedication, absorption), measured using the Utrecht Work Engagement Scale [49]; four dimensions of psychological empowerment (competence, meaning, self-determination, impact), measured using Spreitzer’s Psychological Empowerment Scale [23]; problem solving, measured using an abbreviated version of Heppner’s Problem Solving Inventory [50]; and physical and mental health, measured using the SF-8 Health Survey [51].

#### Organizational context variables

Contextual variables investigated were: adequate orientation (single item on the TREC survey), dementia-related responsive behaviours (six items on the TREC survey), and the 10 scales of the 53-item Alberta Context Tool (ACT). The ACT is a survey designed to measure modifiable elements of organizational context [45]. It is comprised of 10 concepts: leadership, culture, evaluation (feedback of data), social capital, organizational slack (composed of 3 concepts: staff, time, space), formal interactions, informal interactions, and structural and electronic resources. The ACT, originally developed for use in acute care hospitals [52], was modified for and produced reliable and valid scores when used with healthcare aides and registered nurses in residential LTC [53, 54].

### Statistical analysis

We used descriptive statistics to determine the level of job satisfaction among allied healthcare providers, as well as to create a profile of the sample, including demographic, individual, and context variables. Means and standard deviations were used for all interval-level variables and frequency counts and proportions were used for all categorical-level variables. All continuous variables were treated as continuous. All variables that were significantly associated with job satisfaction in the bivariate analyses (*p* ≤ 0.05) were entered into a General Estimating Equation (GEE) model.

Since TREC data have a natural hierarchical structure (staff are nested in facilities that are nested within regions and provinces), and allied healthcare provider responses within a facility may be correlated and clustered, we used GEE modeling to account for any possible data clustering [55]. An extension of the generalized linear model, GEE models are population-level approaches to model longitudinal and/or clustered data [56]. GEE models provide a semi-parametric approach to analysis of longitudinal data and can be used to test main effects and interactions with categorical and continuous independent variables [57, 58]. GEE models have many advantages, including that the covariance structure does not need to be correctly specified to obtain consistent estimates of regression coefficients and standard errors. As well, covariates can have interaction terms or be nonlinear transformations of the original independent variables [57, 58]. In the current study, we derived scale scores using pairwise deletions of missing cases. We derived mean scale scores using all items completed by the participant for the scale. We ran the model using listwise deletion of missing cases. The significance level was set at *p* < .05 for all analyses. We performed all analyses using IBM SPSS Statistics for Mac, v21.0 software (Chicago, IL, USA).

We used the STROBE guideline for reporting observational studies to report the study findings (see Additional file 1) [59].

## Results

### Demographic characteristics

A total of 334 allied health professionals from 77 residential LTC participated in the TREC program (Table 2). Most participants were women (84.1%) and worked in large facilities (54.8%). The largest age group was 50–59 years (26.9%). The most common allied health roles were recreation therapist assistant/attendant/aide (21.0%), recreation therapist (20.1%), rehabilitation therapist (18.0%), and rehabilitation therapist assistant/attendant/aide (15.3%). Social workers (10.2%), dieticians (7.2%), and clinical pharmacists (0.9%) were also represented in our sample. The data were initially compared across the three Canadian provinces. As only two variables were statistically different between the subsamples, the subsamples were deemed comparable and grouped together for further analyses.

**Table 2:** Comparison of demographic characteristics among allied health professionals by province (*N* = 334)

		Province				χ^2^ / ANOVA
		British Columbia *n = 131*	Alberta *n = 162*	Manitoba *n = 41*	Total *n = 334*	*p* value^1^
Owner-operator model [n (%)]	Public not-for-profit	34 (26.0)	23 (14.2)	1 (2.4)	58 (17.4)	**0.003** ^**2**^
	Private for-profit	54 (41.2)	69 (42.6)	18 (43.9)	141 (42.2)	
	Voluntary not-for-profit	43 (32.8)	70 (43.2)	22 (53.7)	135 (40.4)	
	*Missing*	0 (0.0)	0 (0.0)	0 (0.0)	0 (0.0)	
Facility size [n (%)]	Small (≤79 beds)	21 (16.0)	26 (16.0)	4 (9.8)	51 (15.3)	**< 0.001** ^**3**^
	Medium (80–120 beds)	64 (48.9)	25 (15.4)	11 (26.8)	100 (29.9)	
	Large (> 120 beds)	46 (35.1)	111 (68.5)	26 (63.4)	183 (54.8)	
	*Missing*	0 (0.0)	0 (0.0)	0 (0.0)	0 (0.0)	
Gender [n (%)]	Female	111 (84.7)	137 (84.6)	33 (80.5)	281 (84.1)	0.670
	Male	15 (11.5)	17 (10.5)	4 (9.8)	36 (10.8)	
	*Missing*	5 (3.8)	8 (4.9)	4 (9.8)	17 (5.1)	
Age group [n (%)]	< 20 years	0 (0.0)	0 (0.0)	0 (0.0)	0 (0.0)	0.493
	20–29 years	22 (16.8)	37 (22.8)	7 (17.1)	66 (19.8)	
	30–39 years	32 (24.4)	47 (29.0)	7 (17.1)	86 (25.7)	
	40–49 years	29 (22.1)	30 (18.5)	10 (24.4)	69 (20.7)	
	50–59 years	38 (29)	36 (22.2)	16 (39.0)	90 (26.9)	
	60–70 years	9 (6.9)	11 (6.8)	1 (2.4)	21 (6.3)	
	> 70 years	1 (0.8)	1 (0.6)	0 (0.0)	2 (0.6)	
	*Missing*	0 (0.0)	0 (0.0)	0 (0.0)	0 (0.0)	
Highest education [n (%)]	Diploma/certificate	60 (45.8)	55 (34.0)	20 (48.8)	135 (40.4)	**0.012** ^**4**^
	Bachelor’s degree	53 (40.5)	69 (42.6)	14 (34.1)	136 (40.7)	
	Graduate degree	15 (11.5)	28 (17.3)	3 (7.3)	46 (13.8)	
	*Missing*	3 (2.3)	10 (6.2)	4 (9.8)	17 (5.1)	
Current enrolment [n (%)]	Yes	11 (8.4)	11 (6.8)	3 (7.3)	25 (7.5)	0.873
	No	120 (91.6)	151 (93.2)	38 (92.7)	309 (92.5)	
	*Missing*	0 (0.0)	0 (0.0)	0 (0.0)	0 (0.0)	
Primary role [n (%)]	Rehabilitation Therapist	25 (19.1)	32 (19.8)	3 (7.3)	60 (18.0)	**0.041** ^**5**^
	Clinical Pharmacist	0 (0.0)	3 (1.9)	0 (0.0)	3 (0.9)	
	Recreational Therapist	27 (20.6)	26 (16.0)	14 (34.1)	67 (20.1)	
	Social Worker	9 (6.9)	18 (11.1)	7 (17.1)	34 (10.2)	
	Dietician	12 (9.2)	10 (6.2)	2 (4.9)	24 (7.2)	
	Rehabilitation Therapist Assistant/Attendant/Aide	18 (13.7)	27 (16.7)	6 (14.6)	51 (15.3)	
	Recreation Therapist Assistant/Attendant/Aide	30 (22.9)	37 (22.8)	3 (7.3)	70 (21.0)	
	Other	10 (7.6)	9 (5.6)	6 (14.6)	25 (7.5)	
	*Missing*	0 (0.0)	0 (0.0)	0 (0.0)	0 (0.0)	
Hours worked in 2 weeks [Mean (SD)]		53.305 (24.584)	65.165 (19.536)	67.308 (19.113)	60.724 (22.403)	**< 0.001** ^**6**^
Years worked in current role [Mean (SD)]		8.733 (8.841)	8.251 (8.253)	9.415 (8.170)	8.583 (8.457)	0.723
Years worked in facility [Mean (SD)]		5.136 (6.691)	7.179 (8.000)	7.810 (7.439)	6.414 (7.489)	**0.036** ^**7**^

^1^Chi-square test was used to test statistical differences in categorical variables (owner-operator model, facility size, gender, age group, highest education, primary role, current enrolment) and one-way ANOVA was used for quantitative (interval and ratio level variables – hours worked, years worked in current role, years worked in facility)

^2^Post-hoc test used was Bonferroni correction. No significant differences were found

^3^Post-hoc test used was Bonferroni correction. Significant differences were for the following facility size categories: medium (British Columbia compared to Alberta); large (British Columbia compared to Alberta)

^4^Post-hoc test used was Bonferroni correction. No significant differences were found

^5^Post-hoc test used was Bonferroni correction. No significant differences were found

^6^Post-hoc test used was Bonferroni correction. Significant differences were between British Columbia and Alberta, and British Columbia and Manitoba

^7^Post-hoc test used was Bonferroni correction. No significant differences were found

Bolded items indicate a significant relationship at *p* < 0.05

### Bivariate and multivariate analysis

The mean job satisfaction score was 4.20 (*SD* = 0.66). The average scores for study variables are in Table 3. Given the partial conceptual overlap of some variables, a correlation matrix of all the demographic-, individual-, and contextual-level factors is provided (Additional file 2: Table S1). The bivariate analysis showed 22 of 33 (67%) variables identified as potentially important to allied healthcare provider job satisfaction based on past empirical studies and theory were significantly associated with job satisfaction in our sample (Table 4). To determine the sample size required for the GEE model, we used the rule of thumb of a minimum of 10 events per one predictor variable to arrive at a minimum sample size of 220. These variables, along with primary role, were included in the subsequent GEE model (Table 5).

**Table 3:** Average scores for study variables

Category	Variable	*N*	Mean (*SD*)
Dependent Variable	Job Satisfaction	325	4.20 (0.660)
Independent Variables			
Demographic Characteristics	Hours worked in 2 weeks	323	60.72 (22.40)
	Time worked in current role	313	8.58 (8.46)
	Time worked in unit/nursing home	318	6.44 (7.49)
	Year Licensed	281	2002 (11)
Individual-Level Factors	Burnout: Emotional Exhaustion	322	1.85 (1.298)
	Burnout: Cynicism	318	1.67 (1.241)
	Burnout: Efficacy	321	4.74 (0.962)
	Work Engagement: Vigor	324	5.18 (0.911)
	Work Engagement: Dedication	325	5.45 (0.852)
	Work Engagement: Absorption	324	5.68 (0.575)
	Empowerment: Competence	324	4.38 (0.521)
	Empowerment: Meaning	322	4.48 (0.534)
	Empowerment: Self-Determination	324	4.10 (0.810)
	Empowerment: Impact	322	3.60 (0.674)
	Problem Solving	322	3.91 (0.377)
	Physical Health Status	322	51.58 (7.590)
	Mental Health Status	322	49.41 (9.765)
Context-Level Factors	Leadership	332	3.84 (0.853)
	Culture	331	3.95 (0.621)
	Evaluation	333	3.59 (0.774)
	Social Capital	323	3.93 (0.521)
	Organizational Slack-Staffing	325	2.57 (0.963)
	Organizational Slack-Space	320	3.77 (1.021)
	Organizational Slack-Time	320	2.99 (0.682)
	Formal Interactions	323	1.34 (1.274)
	Informal Interactions	314	4.08 (2.272)
	Structure and Electronic Resources	317	3.84 (2.017)
	Adequate Orientation	325	3.73 (0.981)
	Dementia-Related Responsive Behaviours	325	1.80 (1.523)

**Table 4:** Bivariate analysis – allied health professionals (*N* = 334)

	Variable	correlation coefficient	*p* value	Sample Size (*n*)
Demographic Characteristics	Gender		.102^1^	309
	Age group		.771^2^	325
	Highest Education		.123^2^	325
	Current Enrolment		.529^1^	325
	Primary Role		.465^1^	325
	Hours worked in 2 weeks	−.058	.308^3^	315
	Time worked in current role	−.006	.915^3^	304
	Time worked in unit/LTC	.020	.722^3^	309
	Year Licensed	−.050	.408^3^	277
Individual-Level Factors	Burnout: Emotional Exhaustion	−.471^a^	**< .001** ^3^	322
	Burnout: Cynicism	−.548^a^	**< .001** ^3^	318
	Burnout: Efficacy	.292^a^	**< .001** ^3^	321
	Work Engagement: Vigor	.489^a^	**< .001** ^3^	324
	Work Engagement: Dedication	.499^a^	**< .001** ^3^	325
	Work Engagement: Absorption	.391^a^	**< .001** ^3^	324
	Empowerment: Competence	.114^b^	**.040** ^3^	324
	Empowerment: Meaning	.418^a^	**< .001** ^3^	322
	Empowerment: Self-Determination	.495^a^	**< .001** ^3^	324
	Empowerment: Impact	.420^a^	**< .001** ^3^	322
	Problem Solving	.122^b^	**.029** ^3^	321
	Physical Health Status	.055	.327^3^	322
	Mental Health Status	.268^a^	**< .001** ^3^	322
Context-Level Factors	Leadership	.424^a^	**< .001** ^3^	324
	Culture	.572^a^	**< .001** ^3^	323
	Evaluation	.352^a^	**< .001** ^3^	324
	Social Capital	.469^a^	**< .001** ^3^	323
	Organizational Slack-Staffing	.266^a^	**< .001** ^3^	325
	Organizational Slack-Space	.220^a^	**< .001** ^3^	320
	Organizational Slack-Time	.350^a^	**< .001** ^3^	320
	Formal Interactions	.103	.064^4^	323
	Informal Interactions	.149^a^	**.008** ^4^	313
	Structure and Electronic Resources	.170^a^	**.002** ^4^	317
	Adequate Orientation	.348^a^	**< .001** ^3^	325
	Dementia-related Responsive Behaviours	−.071	.200^4^	325

^a^ Mean difference or correlation coefficient is significant at the 0.01 level (2-tailed)

^b^ Mean difference or correlation coefficient is significant at the 0.05 level (2-tailed)

^1^ Independent Samples T-test

^2^ One-way ANOVA

^3^ Pearson’s *r*

^4^ Spearman *rho*

Bolded items indicate a significant relationship at *p* < 0.05

**Table 5:** GEE model: allied health professionals (*N* = 288)

Category	Variables	β	SE	95% CI	*p* value
	Intercept	1.112			.034
Individual-Level Factors	Burnout: Emotional Exhaustion	−.042	.0293	−.100–.015	.150
	Burnout: Cynicism	−.113	.0338	−.179 – -.046	**.001**
	Burnout: Efficacy	.013	.0344	−.054–.081	.699
	Work Engagement: Vigor	.079	.0544	−.028–.185	.148
	Work Engagement: Dedication	.025	.0717	−.116–.165	.732
	Work Engagement: Absorption	.061	.0746	−.085–.207	.414
	Empowerment: Competence	−.224	.0608	−.343 – -.105	**< .001**
	Empowerment: Meaning	.232	.0725	.090–.374	**.001**
	Empowerment: Self-Determination	.128	.0460	.038–.219	**.005**
	Empowerment: Impact	.10	.0448	.022–.197	**.014**
	Problem Solving	.015	.0752	−.132–.163	.839
	Mental Health Status	−.004	.0038	−.011–.004	.318
Context-Level Factors	Leadership	.060	.0394	−.017–.137	.129
	Culture	.097	.0666	−.034–.228	.145
	Evaluation	−.034	.0415	−.115–.047	.414
	Social Capital	.158	.0632	.034–.282	**.012**
	Organizational Slack-Staffing	−.021	.0314	−.082–.041	.511
	Organizational Slack-Space	−.015	.0247	−.064–.033	.538
	Organizational Slack-Time	.096	.0439	.010–.182	**.029**
	Formal Interactions	−.057	.0268	−.110 – -.005	**.033**
	Informal Interactions	−.008	.0141	−.035–.020	.587
	Structural and Electronic Resources	−.009	.0166	−.042–.023	.571
	Adequate Orientation	.088	.0314	.027–.150	**.005**
QIC: 103.181					
QICC: 96.712					

*β* standardized coefficient, *CI* confidence interval, *SE* standard error

Bolded items indicate a significant relationship at *p* < 0.05

In the final GEE model, eight variables predicted job satisfaction; five variables were individual level and the remaining three variables were organizational context concepts. At the individual level, higher burnout-cynicism (β = −.113, *p* = .001) and the competence subscale of psychological empowerment (β = −.224, *p* = < .001) predicted lower job satisfaction. Three components of psychological empowerment were predictive of job satisfaction, with higher empowerment being associated with higher job satisfaction: empowerment-meaning (β = .232, *p* = .001), empowerment-self-determination (β = .128, *p* = .005), and empowerment-impact (β = .110, *p* = .014). Three organizational context concepts predicted job satisfaction: social capital, organizational slack-time, and adequate orientation. Higher scores on organizational slack-time (β = .096, *p* = .029), social capital (β = .158, *p* = .012), and adequate orientation (β = .088, *p* = .005) predicted higher job satisfaction levels.

## Discussion

This study sought to identify factors affecting job satisfaction among a wide variety of allied healthcare providers in residential LTC. Multivariate analysis revealed that five individual-level variables and three organizational context variables predicted allied healthcare provider job satisfaction in LTC. Consistent with previous studies [16, 19], demographic variables were not predictive of allied healthcare provider job satisfaction. Given the limited theoretical evidence in support of empowerment models of job satisfaction and given the similarities between nurses’ and allied health workers’ work environments [35–40], we relied on published studies of factors affecting job satisfaction among RNs and LPNs employed in LTC facilities when examining our findings among allied health staff.

### Individual-level variables

In the present study, five individual-level variables predicted job satisfaction among allied healthcare providers in LTC, with psychological empowerment-competence and burnout-cynicism predicting lower job satisfaction and the meaning, self-determination, and impact subscales of psychological empowerment predicting higher job satisfaction. To the best of our knowledge, this is the first study to show that burnout-cynicism affects allied health professionals’ job satisfaction in LTC.

#### Psychological empowerment

Largely consistent with Spreitzer’s Theory of Psychological Empowerment [21–23], which suggests that high levels of psychological empowerment promote job satisfaction, higher scores on three subscales of Spreitzer’s Psychological Empowerment Scale [23] predicted higher levels of job satisfaction among allied healthcare providers. These findings are in line with previous research on allied health provider job satisfaction in LTC. Greater autonomy [16, 18] and ability to contribute to decision making [17] have long been shown to promote job satisfaction among allied health professionals. For example, Tourangeau and colleagues [60] found that higher job satisfaction among allied healthcare providers was associated with higher psychological empowerment and higher sense of personal accomplishment, and that lower levels of autonomy on the job were positively correlated with intention to leave. As well, studies in diverse workers in varied settings have reliably found that meaning is positively associated with job satisfaction [23, 32, 61, 62]. In the present study, meaning had the largest predictor coefficient among the psychological empowerment components.

Our findings also reflect research on allied healthcare providers working in non-LTC settings. In a study examining aspects of allied healthcare providers’ jobs that contribute to job satisfaction and intention to leave a metropolitan hospital, Wilson and colleagues [63] found a significant negative correlation between factors related to psychological empowerment, including autonomy and accomplishment, and their intention to leave their job. Similarly, in a literature review of factors promoting retention of rural and remote allied health professionals, Campbell and colleagues [64] found that autonomy was a positive intrinsic incentive promoting retention, while not same, is nonetheless related to job satisfaction. These findings support the notion that autonomy to make decisions about how to do one’s work results in a sense of ownership, which may explain why higher psychological empowerment is predictive of higher job satisfaction among allied healthcare providers in the present study.

We found a negative relationship between competence and job satisfaction among allied health staff. These results are surprising, as competence was positively predictive of job satisfaction in the bivariate analysis. Although general suppressor effects, suggested by the negative beta coefficient, are difficult to interpret [65], previous studies have found mixed results for the role of competence in job satisfaction. Among nurses, low levels of competence have been associated with work stress [66], whereas higher levels of competence have been shown to predict job satisfaction [67]. Among nurse aides, training has been shown to improve competence in managing dementia-related behavioral problems, improving job satisfaction [68]. On the other hand, Li and colleagues [32] found that among nurses, competence was not associated with job satisfaction in the bivariate analysis, and only the self-determination subscale of psychological empowerment was predictive of job satisfaction in multivariate analysis. In a different study, only meaning and impact, not competence, were significant predictors of work stress among nurses [31].

Outside of the nursing field, studies examining the relationship between job satisfaction and competence have also been inconclusive. In a study of 174 customer service employees, Carless [61] found that competence was a negative predictor of job satisfaction, whereas meaning and impact were positively predictors of job satisfaction (self-determination was not significant). Spreitzer et al. [23] found that competence was positively correlated with job satisfaction among subordinates but did not affect job satisfaction among supervisors. Since our sample included both registered professionals (i.e., therapists, social workers, dieticians) and their assistants/aides, we examined whether the association between competence and job satisfaction differed between these two groups. We found that opposite relationship to that of Spreitzer et al. [23]: competence was positively correlated with job satisfaction among registered professionals (*r* = .155, *p* = .036, *N* = 184), but had no effect among assistants and aides (*r* = .098, *p* = .282, *N* = 118). Because competence includes the perceived similarity or mismatch between the skills possessed and the skills required for a particular job [62], it may be that significant incongruence in either direction (i.e., underqualified or overqualified) may lead to job dissatisfaction. In our sample, it may be that assistants and aides felt overqualified for their positions, resulting in the negative association between competence and job satisfaction, whereas regulated professionals felt “just right” for the job, leading to a positive relationship.

Conversely, it may be that there are complex interactions between dimensions of psychological empowerment, leading to enhancement of suppression of job satisfaction. For instance, Wang and Lee [62] found that, when examined separately, dimensions of psychological empowerment had positive effects on job satisfaction; however, when examined together, individual dimensions enhanced or suppressed the influence of other dimensions on job satisfaction. These findings suggest that the notion that each component of psychological empowerment has an additive effect on job satisfaction is too simplistic and incomplete.

Given these findings and the findings of the current study, further research exploring the causal pathway between empowerment and job satisfaction is needed. Specifically, a quantitative study with a large sample of both regulated professionals and assistants/aides would permit researchers to develop two separate models of job satisfaction to untangle differences and similarities between regulated professionals and their assistants/aides. As well, there is a need to conduct qualitative studies to further examine the relationship between perception of one’s competence for the job and job satisfaction among allied health staff in LTC facilities.

Leaders and managers within facilities can empower staff, thereby enhancing their job satisfaction. According to Gleasonwynn and Mindel [16], allied health professionals who know their role responsibilities and how to accomplish their duties should be given freedom and flexibility when making decisions regarding how to do their job. Organizations should provide information about their mission and values to provide staff with a sense of purpose and meaning in their work [23]. In addition, a program that fosters communication and feedback to staff may help staff feel valued and supported [23]. LTC facilities could also provide incentives for taking part in the decision-making process to empower employees to take control of their work [69]. For example, allowing allied health staff to take part in flexible scheduling would also provide employees with more control and autonomy over their work life, thereby likely enhancing their job satisfaction [70]. Managers who provide staff with a sense of autonomy could also promote empowerment, making staff more likely to stay on the job [70]. As the meaning dimension of empowerment appears to have a consistent positive effect on job satisfaction [62], managers should emphasize programs that increase staff’s sense of meaning. However, given the potential complex interactions between dimensions of psychological empowerment and job satisfaction, managers should be careful to ensure that their policies do not inadvertently work against employees’ job satisfaction.

#### Burnout-cynicism

In the present study, the cynicism dimension of burnout predicted lower job satisfaction. Cynicism measures the indifference or a distant attitude that individuals may feel toward their work. To the best of our knowledge, this is the first study to identify cynicism as a factor affecting job satisfaction among allied health professionals in LTC facilities. However, studies have found that other dimensions of burnout are associated with reduced job satisfaction among allied health professionals. In a study of factors affecting job satisfaction in LTC facilities among allied health workers, Tourangeau et al. [60] found that higher burnout-exhaustion, which measures overextended and exhausted by one’s work, and higher burnout-depersonalization, which measures an impersonal response toward recipients of one’s service, care treatment, or instruction, were associated with lower job satisfaction.

The relatively limited time allied healthcare providers spend with residents in LTC may contribute to their perception of cynicism regarding their ability to effectively meet patients’ needs. In addition, allied health professionals in LTC facilities tend to focus on delaying onset of immobility and easing discomfort as opposed to therapeutic progress for physical therapy and related concerns, which may further contribute to allied healthcare providers sense that they may not be putting in enough time [71]. Indeed, Balogun et al. [71] argued that poor treatment outcomes in working with individuals with chronic conditions, lack of organizational slack time, lack of peer and supervisors’ support, inadequate resources, and interprofessional conflicts contribute to burnout among allied health professionals. Balogun et al. [71] found significant relationships between allied healthcare providers relationships with peers and supervisors and burnout. They found that support from supervisors explained 7% of the variance in emotional exhaustion and support from colleagues accounted for 9.6% of the variance in cynicism. Thus, the requirements for emotional labour may be lower for allied healthcare providers but frustration and cynicism due to prolonged and poor treatment outcomes may be higher, reducing the impact of emotional exhaustion on their job satisfaction while increasing the impact of cynicism. Taken together, these findings suggest that burnout-cynicism is an important factor affecting job satisfaction among allied healthcare providers in LTC facilities that needs addressing.

#### Work engagement

Consistent with previous studies [72, 73], work engagement was correlated with job satisfaction in the present study. In a study of health care professionals employed in a community setting, Noblet et al. [72] found a significant correlation between work satisfaction and work engagement. In the current study, all components of work engagement were likewise correlated with job satisfaction in the bivariate analysis. In the final model, however, work engagement was not a significant predictor of job satisfaction. Given the significant negative correlation between each component of work engagement and the exhaustion and cynicism components of burnout, which is consistent with the conceptualization of work engagement as being the “opposite” of burnout [74], it is possible that some of the predicted variance attribute to work engagement components becoming attributed to burnout-cynicism, reducing the overall significance of work engagement in the final model. However, we were unable to identify studies that examined the relationship between work engagement and job satisfaction among allied health professionals in LTC facilities. Thus, further research to examine the role of work engagement in job satisfaction among allied health staff in LTC facilities is warranted.

### Organizational context variables

In the present study, three context variables positively predicted job satisfaction among allied healthcare providers in LTC: social capital, organizational slack-time, and adequate orientation. To the best of our knowledge, this is the first study to show organizational slack-time and adequate orientation affect job satisfaction among allied healthcare staff in LTC facilities.

#### Social capital

The present study identified social capital as significant positive predictor of job satisfaction among allied health professionals. This is in line with Kanter’s [20] theory of structural conditions necessary for job empowerment and job satisfaction, which proposes that informal power derived from relationships with superiors, peers, and subordinates promotes job satisfaction. In addition, these findings are consistent with previous studies. For instance, Wilson et al. [63] and Rodwell, Noblet, Demire, and Steane [75] found that communication and support from the manager were significantly correlated with intention to stay and job satisfaction among allied healthcare providers; and Gleasonwynn and Mindel [16] who found that peer support, emotional support, and morale enhancement improved job satisfaction among LTC allied healthcare providers. Finally, Stagnitti, Schoo, Dunbar, and Reid [76] also found that when allied healthcare providers felt supported in the workplace, they expressed higher intention to stay.

The importance of communication among colleagues may be particularly salient for allied healthcare providers, given that there are relatively few allied health professionals employed in a single LTC facility compared to other care provider groups and that allied healthcare providers tend to move around units or floors in a facility more than other care providers, leaving limited opportunity for peer support [16]. Consistent with this, Collins et al. [69] found that job satisfaction depended on participants’ integration within their own professional group and with colleagues in other fields.

The concept of *esprit de corp*, defined as “feelings of group belonging and solidarity derived from a sense of the position nurses occupy as a subordinate collective within the healthcare system” [77], may also explain the importance of social capital for allied healthcare providers. Miller et al. [77] found that feelings of professional solidarity among nurses were interpreted negatively by other professionals. As a result, social capital, which may indicate the extent to which allied healthcare providers are integrated into the facility and the extent of interprofessional collegiality, may be more important than unit or facility culture for this population. For allied healthcare providers, who may be excluded from the *esprit de corps* of the nursing groups [77], integration and interprofessional communication may play a particularly important role in job satisfaction. Taken together, the present findings suggest that support provided through social connections and social capital among allied healthcare providers and between allied healthcare providers and nursing staff may enable the former to receive better emotional support, boosting morale and job satisfaction.

In the present study, social capital was defined as the stock of active connections among colleagues. Social capital has also been defined as including “the norms and networks that enable people to act collectively” as well as “how people interact with each other (and on what terms)” [78]. A concept analysis of social capital identified “networks of social relationships at work,” “shared assets,” and “shared ways of knowing,” which includes an organization’s culture based on values, beliefs, and understandings, as key features of social capital [79, 80]. Given these broad definitions of social capital, it is not surprising that social capital was also correlated with culture and informal interactions, which may be categorized under “social integration” (i.e., “connectedness with or caring about fellow workers”) [81]. Social capital was also positively correlated with leadership. While these variables were significantly correlated with job satisfaction in the bivariate analysis, only social capital was found to be a significantly predictive of job satisfaction in the final model. It may be that social capital explains more of the variance in job satisfaction than either culture, leadership, and informal interactions [82]. McColskey [81] argued that good leaders create a general ambience on a unit through interactions of many factors that in turn increase social capital. Thus, it may be that good leadership promotes a positive work culture, which then contributes to an environment where allied health staff experience a positive social capital, to which they then attribute their job satisfaction. From the perspective of employees, social capital may be the most proximal factor affecting job satisfaction.

#### Organizational slack-time

In the present study, perceptions of a lack of organizational slack - time, which refers to the perceived availability of time to provide resident care and share best practice knowledge, was a predictor of lower job satisfaction among allied healthcare providers. While, to the best of our knowledge, organizational slack-time has not been assessed in any of the previous studies on allied healthcare provider job satisfaction, it may be that limited time with each patient contributes to job dissatisfaction by increasing burnout-cynicism resulting from insufficient time to complete job duties, particularly given the long timescales that may be required for treatment among this population [71]. Kanter [20] argues that access to resources, including time to accomplish organizational goals, is an important component of job satisfaction. Our results with respect to slack-time are also consistent with research showing that insufficient time with residents increases stress, which may contribute to poorer job satisfaction. For instance, in a qualitative study of retention among allied health workers in rural areas, O’Toole, Schoo, and Hernan [83] found limited time to do administrative work reduced healthcare providers’ intention to stay. Our findings, in conjunction with past research in the field, suggests increasing allied health care providers organizational slack-time as a means of promoting their job satisfaction.

#### Adequate orientation

The present study also identified adequate orientation as a significant predictor of allied healthcare provider job satisfaction. Adequate job orientation refers to an individual’s perception of whether they have had enough on-the-job orientation and training to carry out their job safely and effectively. Kanter [20], in the Theory of Structural Empowerment, posits that access to information (e.g., technical knowledge, data, expertise) to function effectively in one’s position is important to job satisfaction. Though not directly assessing job satisfaction, Stagnitti et al. [76] found that allied health professionals who were given orientation to their primary position were more likely to express intention to stay longer in the job. It is possible that the lack of adequate job orientation contributed to lower job satisfaction by increasing stress and time required to work out proper protocols. We recently conducted a similar analysis on nurses (Aloisio et al. :Individual and organizational predictors of nurses' job satisfaction in residential long term care, submitted) and found that adequate orientation was also a significant predictor of job satisfaction among regulated nurses. Among nurses, perception of inadequate training leaves nurses feeling unprepared and overwhelmed about their responsibilities [84, 85], and lack of clarity about one’s role contributes to confusion [86], adversely affecting job satisfaction. Among allied health professionals, adequate orientation is associated with intention to stay employed in the current facility [70]. Likewise, lack of adequate orientation is associated with increased stress, as staff are forced to spend more time identifying appropriate contacts and proper protocols for organizational procedures [87].

Given the relationship between adequate orientation and job satisfaction identified in this and previous studies, orientation and training programs need to be developed that address the needs of allied health professionals employed in LTC facilities. In a systematic review of the effectiveness of training program aimed at health professionals working with older individuals with dementia, McCabe et al. [84] found that training and education programs reduced staff stress levels, improved employees’ sense of self-worth and satisfaction with work, and reduced turnover. Training has also been shown to reduce staff members’ perception of the severity and frequency of dementia-related behaviors among staff [88], reducing job strain and enhancing job satisfaction. In a study of factors affecting job satisfaction among nurses and allied health professionals, Collins and colleagues [69] found that respondents who felt adequately trained and prepared for their roles reported higher job satisfaction. The authors posited that training made staff feel more capable and comfortable at their jobs.

Summarizing the literature on education and training programs for staff employed in LTC facilities, Moyle et al. [89] provided recommendations for orientation and training programs based on their findings. Among the recommendations, Moyle et al. [89] suggest involving the target audience in planning and design of the information to ensure that the information provided matches the needs of the audience. Input from staff would allow the organization to empower staff to gain the resources they feel are most important to them. Therefore, a needs assessment by individual LTC facilities could provide clarity into what may be missing from the current orientation provided. This is particularly important as the design of the program plays a key role in program success [90]. Moreover, to ensure success, the curricula of the training and orientation programs should also be embedded in the culture of the LTC facility [91]. As well, effective leaders that are supportive of training and education initiatives are crucial to program success [89]. To promote job satisfaction among staff, leaders and change agents within the organization need to ensure consistent and ongoing commitment to developing and implementing orientation and training programs that meet the needs of the staff [89]. By providing adequate orientation, facilities can address multiple factors shown to influence job satisfaction in the present study.

### Strengths and limitations

#### Strengths

The study has important strengths. It is the first study to evaluate, using multivariate analysis, a large variety of factors, individual and organizational, affecting job satisfaction among a relatively large sample of allied health care providers in residential LTC settings. Data was collected from a random stratified sample of LTC facilities using well-validated responses with a high degree of quality assurance processes [92]. Additionally, secondary analyses, such as this one, are time- and cost-effective, as resources do not need to be expended on collecting data. They also serve as a useful exploratory tool.

#### Limitations

The cross-sectional nature of our analysis limits ability to assess whether temporal changes in organizational context affect allied healthcare providers’ job satisfaction. Further, as this was a secondary analysis, the data did not contain all of the variables of interest identified in the literature, e.g., levels of administrative duties [17], contextual variables such as less routinization [18], satisfaction with residents and residents’ families [16], pay and benefits (particularly as compared to those working in other sectors) [93–95], and opportunities for promotion and career advancement [18, 96]. Additionally, some of the scales used in the original study with established reliability, when administered to healthcare aides [54] and nurses [53] in LTC, displayed lower alpha scores of <.70 when administered to allied healthcare providers in our study. Thus, future research refining the TREC survey for allied healthcare providers is warranted. Additional limitations include the self-reported nature of all the data, including contextual factors.

## Conclusions

The results of the present study suggest that both individual-level and organizational context variables predict job satisfaction among allied healthcare providers in residential LTC facilities in Western Canada. Consistent with Spreitzer’s Theory of Psychological Empowerment, high levels of psychological empowerment were predictive of job satisfaction in the present study. Our findings suggest that efforts to provide adequate orientation and improve social capital and organizational slack-time to allied healthcare providers hold potential to improve their job satisfaction, which may result in lower staff turnover and better quality of care to residents. Future research is needed to identify casual pathways leading to improved job satisfaction. Future studies should also examine strategies to improve the modifiable context factors identified in this study and then evaluate whether those strategies lead to improvements in job satisfaction among allied healthcare providers in residential LTC facilities.

## Additional files

Additional file 1: STROBE (Strengthening The Reporting of OBservational studies in Epidemiology) Checklist. This file contains the completed STROBE checklist for the present manuscript. (PDF 425 kb)

Additional file 2: Correlation Matrix for Dependent and Independent Study Variables. This file contains a correlation matrix of the dependent variables (demographic-level, individual-level, and context-level variables) and the independent variable (job satisfaction). (DOCX 32 kb)

## Abbreviations

ACT: Alberta context tool
GEE: Generalized estimating equations
LTC: Long-term care
TREC: Translating research in elder care
