# Supplementary material for: Individual and organizational predictors of allied healthcare providers’ job satisfaction in residential long-term care
Source: BMC Health Serv Res. 2018 Jun 25;18:491. doi: 10.1186/s12913-018-3307-3 (PMC6019323; doi:10.1186/s12913-018-3307-3)
Supplement: Supplementary file 2 — Correlation Matrix for Dependent and Independent Study Variables. This file contains a correlation matrix of the dependent variables (demographic-level, individual-level, and context-level variables) and the independent variable (job satisfaction). (DOCX 32 kb) [file 12913_2018_3307_MOESM2_ESM.docx]

**ADDITIONAL FILE 2**

**Table S1. Correlation Matrix for Dependent and Independent Study Variables**

|  | **1** | **2** | **3** | **4** | **5** | **6** | **7** | **8** | **9** | **10** | **11** | **12** | **13** | **14** | **15** | **16** | **17** | **18** | **19** | **20** | **21** | **22** | **23** | **24** | **25** | **26** | **27** | **28** | **29** |
| --- | --- | --- | --- | --- | --- | --- | --- | --- | --- | --- | --- | --- | --- | --- | --- | --- | --- | --- | --- | --- | --- | --- | --- | --- | --- | --- | --- | --- | --- |
| ***Demographic-level variables*** | | | | | | | | | | | | | | | | | | | | | | | | | | | | | |
| 1. Hours worked in  2 weeks | — |  |  |  |  |  |  |  |  |  |  |  |  |  |  |  |  |  |  |  |  |  |  |  |  |  |  |  |  |
| 2. Time worked in current role | -.166^**^ | — |  |  |  |  |  |  |  |  |  |  |  |  |  |  |  |  |  |  |  |  |  |  |  |  |  |  |  |
| 3. Time worked in nursing home | .092 | .466^**^ | — |  |  |  |  |  |  |  |  |  |  |  |  |  |  |  |  |  |  |  |  |  |  |  |  |  |  |
| 4. Year licensed | .145^*^ | -.682^**^ | -.466^**^ | — |  |  |  |  |  |  |  |  |  |  |  |  |  |  |  |  |  |  |  |  |  |  |  |  |  |
| ***Individual-level variables*** | | | | | | | | | | | | | | | | | | | | | | | | | | | | | |
| 5. Burnout: Exhaustion | .134^*^ | -.109 | -.080 | .135^*^ | — |  |  |  |  |  |  |  |  |  |  |  |  |  |  |  |  |  |  |  |  |  |  |  |  |
| 6. Burnout: Cynicism | .167^**^ | -.039 | .018 | .064 | .650^**^ | — |  |  |  |  |  |  |  |  |  |  |  |  |  |  |  |  |  |  |  |  |  |  |  |
| 7. Burnout: Efficacy | .097 | -.017 | -.037 | .009 | -.210^**^ | -.182^**^ | — |  |  |  |  |  |  |  |  |  |  |  |  |  |  |  |  |  |  |  |  |  |  |
| 8. Eng.: Vigor | .055 | .038 | -.008 | -.100 | -.540^**^ | -.423^**^ | .506^**^ | — |  |  |  |  |  |  |  |  |  |  |  |  |  |  |  |  |  |  |  |  |  |
| 9. Eng.: Dedication | .047 | -.034 | -.061 | -.017 | -.437^**^ | -.466^**^ | .446^**^ | .728^**^ | — |  |  |  |  |  |  |  |  |  |  |  |  |  |  |  |  |  |  |  |  |
| 10. Eng.: Absorption | .023 | -.006 | -.052 | -.060 | -.298^**^ | -.326^**^ | .429^**^ | .640^**^ | .707^**^ | — |  |  |  |  |  |  |  |  |  |  |  |  |  |  |  |  |  |  |  |
| 11. Emp.: Competence | .038 | .098 | .085 | -.143^*^ | -.131^*^ | -.079 | .423^**^ | .236^**^ | .151^**^ | .196^**^ | — |  |  |  |  |  |  |  |  |  |  |  |  |  |  |  |  |  |  |
| 12. Emp.: Meaning | .088 | .039 | .038 | -.116 | -.277^**^ | -.274^**^ | .464^**^ | .400^**^ | .440^**^ | .369^**^ | .536^**^ | — |  |  |  |  |  |  |  |  |  |  |  |  |  |  |  |  |  |
| 13. Emp.: Self-Determination | -.067 | -.028 | -.058 | -.016 | -.310^**^ | -.297^**^ | .099 | .289^**^ | .257^**^ | .165^**^ | .175^**^ | .313^**^ | — |  |  |  |  |  |  |  |  |  |  |  |  |  |  |  |  |
| 14. Emp.: Impact | .101 | .030 | .036 | -.017 | -.289^**^ | -.295^**^ | .293^**^ | .359^**^ | .356^**^ | .264^**^ | .314^**^ | .349^**^ | .400^**^ | — |  |  |  |  |  |  |  |  |  |  |  |  |  |  |  |
| 15. Problem Solving | .004 | .069 | -.073 | -.078 | .073 | -.057 | .272^**^ | .060 | .007 | .104 | .366^**^ | .296^**^ | .173^**^ | .138^*^ | — |  |  |  |  |  |  |  |  |  |  |  |  |  |  |
| 16. Physical Health Status | -.079 | .020 | -.140^*^ | .068 | -.362^**^ | -.187^**^ | .101 | .208^**^ | .072 | .062 | .054 | .083 | .060 | -.032 | .042 | — |  |  |  |  |  |  |  |  |  |  |  |  |  |
| 17. Mental Health Status | -.128^*^ | .049 | .037 | -.090 | -.582^**^ | -.467^**^ | .158^**^ | .382^**^ | .313^**^ | .233^**^ | .115^*^ | .056 | .198^**^ | .148^**^ | -.054 | .200^**^ | — |  |  |  |  |  |  |  |  |  |  |  |  |
| ***Context-level variables*** | | | | | | | | | | | | | | | | | | | | | | | | | | | | | |
| 18. ACT Leadership | .013 | -.004 | -.001 | .094 | -.259^**^ | -.283^**^ | .060 | .264^**^ | .235^**^ | .190^**^ | .015 | .089 | .392^**^ | .221^**^ | .040 | -.002 | .229^**^ | — |  |  |  |  |  |  |  |  |  |  |  |
| 19. ACT Culture | -.004 | -.008 | -.076 | .049 | -.344^**^ | -.446^**^ | .177^**^ | .412^**^ | .410^**^ | .337^**^ | .070 | .247^**^ | .514^**^ | .375^**^ | .142^*^ | .064 | .285^**^ | .579^**^ | — |  |  |  |  |  |  |  |  |  |  |
| 20. ACT Evaluation | .190^**^ | -.122^*^ | -.044 | .171^**^ | -.200^**^ | -.234^**^ | .223^**^ | .324^**^ | .357^**^ | .284^**^ | .101 | .166^**^ | .285^**^ | .336^**^ | .109 | .063 | .164^**^ | .391^**^ | .557^**^ | — |  |  |  |  |  |  |  |  |  |
| 21. ACT Social Capital | .015 | .079 | -.021 | .015 | -.221^**^ | -.352^**^ | .216^**^ | .245^**^ | .195^**^ | .162^**^ | .110^*^ | .237^**^ | .400^**^ | .275^**^ | .258^**^ | .097 | .131^*^ | .442^**^ | .581^**^ | .427^**^ | — |  |  |  |  |  |  |  |  |
| 22. ACT OS: Staffing | .044 | -.097 | -.095 | .073 | -.274^**^ | -.215^**^ | .130^*^ | .201^**^ | .161^**^ | .122^*^ | .097 | .080 | .201^**^ | .194^**^ | .082 | .146^**^ | .223^**^ | .236^**^ | .307^**^ | .232^**^ | .192^**^ | — |  |  |  |  |  |  |  |
| 23. ACT OS: Space | .121^*^ | .045 | -.126^*^ | .049 | -.069 | -.132^*^ | .126^*^ | .172^**^ | .176^**^ | .209^**^ | .104 | .128^*^ | .178^**^ | .163^**^ | .139^*^ | -.002 | .035 | .237^**^ | .325^**^ | .312^**^ | .325^**^ | .236^**^ | — |  |  |  |  |  |  |
| 24. ACT OS: Time | .063 | .036 | .007 | -.025 | -.200^**^ | -.199^**^ | .191^**^ | .247^**^ | .240^**^ | .196^**^ | .124^*^ | .249^**^ | .295^**^ | .255^**^ | .174^**^ | .043 | .089 | .163^**^ | .284^**^ | .302^**^ | .308^**^ | .292^**^ | .317^**^ | — |  |  |  |  |  |
| 25. ACT Formal Interactions | .292^**^ | .050 | -.029 | -.006 | -.148^**^ | -.112^*^ | .083 | .118^*^ | .138^*^ | .123^*^ | -.006 | .106 | .295^**^ | .137^*^ | .131^*^ | .211^**^ | .061 | .212^**^ | .208^**^ | .326^**^ | .326^**^ | .033 | .333^**^ | .282^**^ | — |  |  |  |  |
| 26. ACT Informal Interactions | .197^**^ | .041 | .055 | -.019 | -.107 | -.110 | .147^**^ | .205^**^ | .181^**^ | .160^**^ | .032 | .152^**^ | .197^**^ | .208^**^ | .125^*^ | .050 | .019 | .152^**^ | .156^**^ | .275^**^ | .286^**^ | .054 | .310^**^ | .351^**^ | .586^**^ | — |  |  |  |
| 27. ACT Structural & Electronic Resources | .216^**^ | -.024 | -.021 | .058 | -.137^*^ | -.181^**^ | .100 | .135^*^ | .210^**^ | .134^*^ | .050 | .154^**^ | .210^**^ | .209^**^ | .162^**^ | .108 | .071 | .124^*^ | .192^**^ | .230^**^ | .203^**^ | .066 | .284^**^ | .423^**^ | .502^**^ | .530^**^ | — |  |  |
| 28. Adequate orientation | .073 | .078 | .103 | -.125^*^ | -.162^**^ | -.208^**^ | .250^**^ | .194^**^ | .234^**^ | .105 | .293^**^ | .258^**^ | .201^**^ | .288^**^ | .053 | .060 | .122^*^ | .198^**^ | .296^**^ | .248^**^ | .168^**^ | .292^**^ | .129^*^ | .215^**^ | .055 | .123^*^ | .076 | — |  |
| 29. Aggression  Towards Staff | .191^**^ | -.062 | .040 | .111 | .156^**^ | .147^**^ | .150^**^ | -.009 | .006 | -.022 | .096 | .017 | -.033 | .026 | .069 | -.078 | -.109^*^ | -.039 | -.062 | .054 | -.086 | -.031 | .050 | .041 | .036 | .176^**^ | .136^*^ | .006 | — |
| 30. Job Satisfaction | -.058 | -.006 | .020 | -.050 | -.471^**^ | -.549^**^ | .293^**^ | .489^**^ | .499^**^ | .391^**^ | .114^*^ | .418^**^ | .495^**^ | .420^**^ | .122^*^ | .055 | .268^**^ | .424^**^ | .572^**^ | .352^**^ | .469^**^ | .266^**^ | .220^**^ | .350^**^ | .103 | .149^**^ | .170^**^ | .348^**^ | -0.055 |

*Note.* Abbreviations: ACT, Alberta Context Tool; Emp, Psychological Empowerment; Eng, Work Engagement; OS, Organizational Slack. All correlations are Pearson's *r* except for #25, 26, 27, and 29, which are Spearman's *rho*. *. Correlation is significant at the 0.05 level (2-tailed). **. Correlation is significant at the 0.01 level (2-tailed).
